# Supplementary material for: Causal interplay between lactose intolerance and gut microbiota: a combined bidirectional Mendelian randomization and in vivo validation study
Source: Front Nutr. 2026 Jun 1;13:1803337. doi: 10.3389/fnut.2026.1803337 (PMC13265576; doi:10.3389/fnut.2026.1803337)
Supplement: Supplementary file 1 [file Data_Sheet_1.zip › supplementary materials/Forward/leave one out cross validation/ebi-a-GCST90027570.finngen_R12_E4_LACTONAS.pdf]

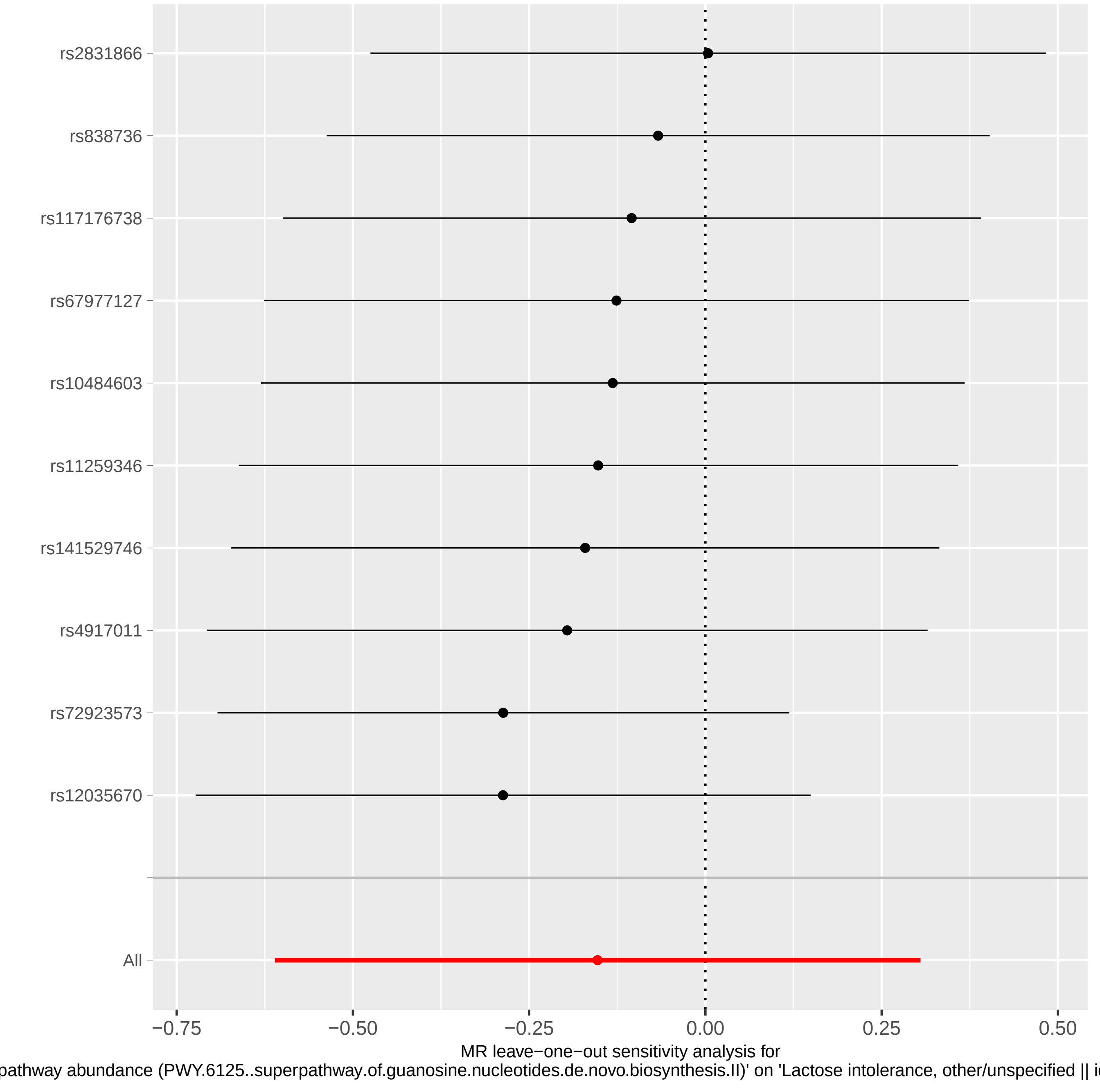
rs2831866
rs838736
rs117176738
rs67977127
rs10484603
rs11259346
rs141529746
rs4917011
rs72923573
rs12035670
All
−0.75
−0.50
−0.25
0.00
0.25
0.50
MR leave−one−out sensitivity analysis for
pathway abundance (PWY.6125..superpathway.of.guanosine.nucleotides.de.novo.biosynthesis.II)' on 'Lactose intolerance, other/unspecified || i
